# Supplementary material for: MethylResolver—a method for deconvoluting bulk DNA methylation profiles into known and unknown cell contents
Source: Commun Biol. 2020 Aug 3;3:422. doi: 10.1038/s42003-020-01146-2 (PMC7400544; doi:10.1038/s42003-020-01146-2)
Supplement: Supplementary file 2 — Reporting summary [file 42003_2020_1146_MOESM2_ESM.pdf]

nature research

Corresponding author(s): Dr. Douglas Arneson and Dr. Kai Wang

Last updated by author(s): Jun 27, 2020

Reporting Summary

Nature Research wishes to improve the reproducibility of the work that we publish. This form provides structure for consistency and transparency in reporting. For further information on Nature Research policies, see Authors & Referees and the Editorial Policy Checklist.

Statistics

For all statistical analyses, confirm that the following items are present in the figure legend, table legend, main text, or Methods section.

n/a	Confirmed	
☐	☒	The exact sample size (*n*) for each experimental group/condition, given as a discrete number and unit of measurement
☐	☒	A statement on whether measurements were taken from distinct samples or whether the same sample was measured repeatedly
☐	☒	The statistical test(s) used AND whether they are one- or two-sided<br>*Only common tests should be described solely by name; describe more complex techniques in the Methods section.*
☐	☒	A description of all covariates tested
☐	☒	A description of any assumptions or corrections, such as tests of normality and adjustment for multiple comparisons
☐	☒	A full description of the statistical parameters including central tendency (e.g. means) or other basic estimates (e.g. regression coefficient) AND variation (e.g. standard deviation) or associated estimates of uncertainty (e.g. confidence intervals)
☐	☒	For null hypothesis testing, the test statistic (e.g. *F*, *t*, *r*) with confidence intervals, effect sizes, degrees of freedom and *P* value noted<br>*Give P values as exact values whenever suitable.*
☒	☐	For Bayesian analysis, information on the choice of priors and Markov chain Monte Carlo settings
☒	☐	For hierarchical and complex designs, identification of the appropriate level for tests and full reporting of outcomes
☐	☒	Estimates of effect sizes (e.g. Cohen's *d*, Pearson's *r*), indicating how they were calculated

Our web collection on statistics for biologists contains articles on many of the points above.

Software and code

Policy information about availability of computer code

Data collection	No software was used to collect the data -- publicly available data was used.
Data analysis	Custom code used to analyze the data in the study is available at: https://github.com/darneson/MethylResolver. All other software used in the analysis of the data was open source R packages which are detailed in the Methods section of the manuscript.

For manuscripts utilizing custom algorithms or software that are central to the research but not yet described in published literature, software must be made available to editors/reviewers. We strongly encourage code deposition in a community repository (e.g. GitHub). See the Nature Research guidelines for submitting code & software for further information.

Data

Policy information about availability of data

All manuscripts must include a data availability statement. This statement should provide the following information, where applicable:

- Accession codes, unique identifiers, or web links for publicly available datasets
- A list of figures that have associated raw data
- A description of any restrictions on data availability

Any data not present in the manuscript or supplementary materials is available from the authors upon reasonable request.

Field-specific reporting

Please select the one below that is the best fit for your research. If you are not sure, read the appropriate sections before making your selection.

☒ Life sciences ☐ Behavioural & social sciences ☐ Ecological, evolutionary & environmental sciences

Life sciences study design

All studies must disclose on these points even when the disclosure is negative.

Sample size	Same size was determined based on the amount of publicly available data. For simulations, sample size was determined based on reasonable permutations/compute time.
Data exclusions	No data was excluded from the analyses.
Replication	For statistical analyses and data simulated data generation, results were repeated with mutiple seeds to ensure findings were reproducible. Machine learning models were built with k-fold cross-validation and leave-one-out analysis.
Randomization	We used publicly available data, samples/participants were already allocated into groups in prior studies.
Blinding	Investigators were blinded to group allocation during data analysis.

Reporting for specific materials, systems and methods

We require information from authors about some types of materials, experimental systems and methods used in many studies. Here, indicate whether each material, system or method listed is relevant to your study. If you are not sure if a list item applies to your research, read the appropriate section before selecting a response.

Materials & experimental systems

n/a	Involved in the study
☒	☐ Antibodies
☒	☐ Eukaryotic cell lines
☒	☐ Palaeontology
☒	☐ Animals and other organisms
☒	☐ Human research participants
☒	☐ Clinical data

Methods

n/a	Involved in the study
☒	☐ ChIP-seq
☒	☐ Flow cytometry
☒	☐ MRI-based neuroimaging
