# Supplementary material for: MethylResolver—a method for deconvoluting bulk DNA methylation profiles into known and unknown cell contents
Source: Commun Biol. 2020 Aug 3;3:422. doi: 10.1038/s42003-020-01146-2 (PMC7400544; doi:10.1038/s42003-020-01146-2)
Supplement: Supplementary file 3 — Description of Additional Supplementary Files [file 42003_2020_1146_MOESM3_ESM.pdf]

Supplementary Data Legends

Supplementary Data 1. *In vitro* mixtures cell fractions ground truth.

Supplementary Data 2. Leukocyte signature matrix, including CpGs used in the signature matrix as well as the Beta value for each cell type across these CpGs.

Supplementary Data 3. Samples used to build the leukocyte methylation reference signature matrix in MethylResolver with ages and ethnicities of individuals indicated.

Supplementary Data 4. Ground truth fractions from reconstructed mixtures of purified human leukocytes and FACS fractions from adult human whole blood.

Supplementary Data 5. Human whole blood true-positive samples used for determining significance threshold and false-positive rate.

Supplementary Data 6. Human primary tissue and iPSC true negative samples used for determining significance threshold and false-positive rate.

Supplementary Data 7. MethylResolver pan-cancer deconvolution of 9,756 cancer samples from TCGA with relative leukocyte subset fractions and estimated tumor purity.

Supplementary Data 8. MethylResolver pan-cancer deconvolution of 9,756 cancer samples from TCGA with tumor purity-scaled leukocyte subset fractions and estimated tumor purity. Tumor purity-scaled leukocyte subset fractions and tumor purity were not inferred for hematologic cancers.

Supplementary Data 9. Spearman correlations of known cancer metrics with relative leukocyte subset fractions for each cancer type considering only significant deconvolutions.

Supplementary Data 10. Spearman correlations of known cancer metrics with tumor purity-scaled leukocyte subset fractions for each cancer type considering only significant deconvolutions.

Supplementary Data 11. Solid tumor cell line panel used to remove cancer-specific CpGs from candidate CpG list in building the MethylResolver reference signature matrix.

Supplementary Data 12. Primary human tissue panel used to remove tissue-specific CpGs from candidate CpG list in building the MethylResolver reference signature matrix
