# Supplementary material for: MethylResolver—a method for deconvoluting bulk DNA methylation profiles into known and unknown cell contents
Source: Commun Biol. 2020 Aug 3;3:422. doi: 10.1038/s42003-020-01146-2 (PMC7400544; doi:10.1038/s42003-020-01146-2)
Supplement: Supplementary file 4 — Supplementary Information [file 42003_2020_1146_MOESM4_ESM.pdf]

## Supplementary Figures

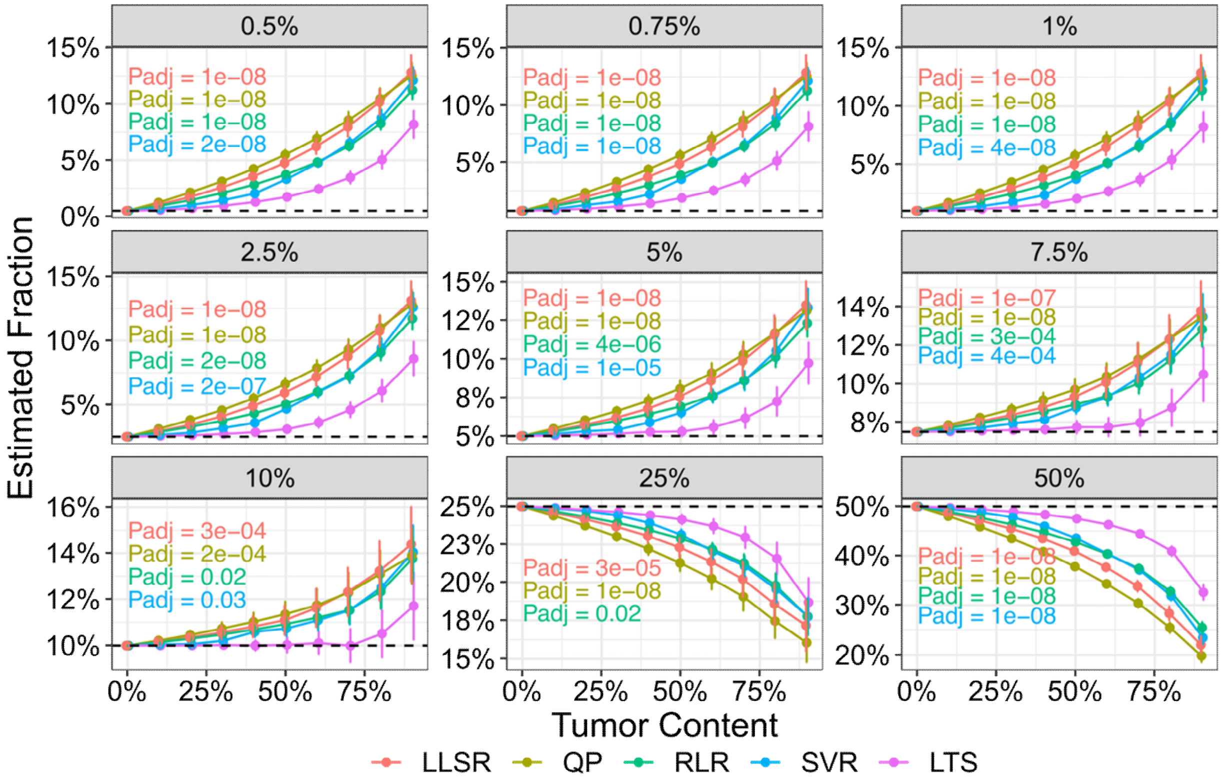


**Supplementary Figure 1.** Benchmarking five different deconvolution methods using *in silico* spike-in experiment. Line color corresponds to the deconvolution method, y-axis is the estimated leukocyte fraction, x-axis is the amount of unknown/tumor content in the mixture, and each panel corresponds to the known spike-in amount of a particular leukocyte with the dotted line being the ground truth. Statistical significance of the performance between MethylResolver and other models was determined using post-hoc pairwise comparisons of two-way ANOVA and significant differences are indicated with the color of the text corresponding to the model. Error bars are the SEM.

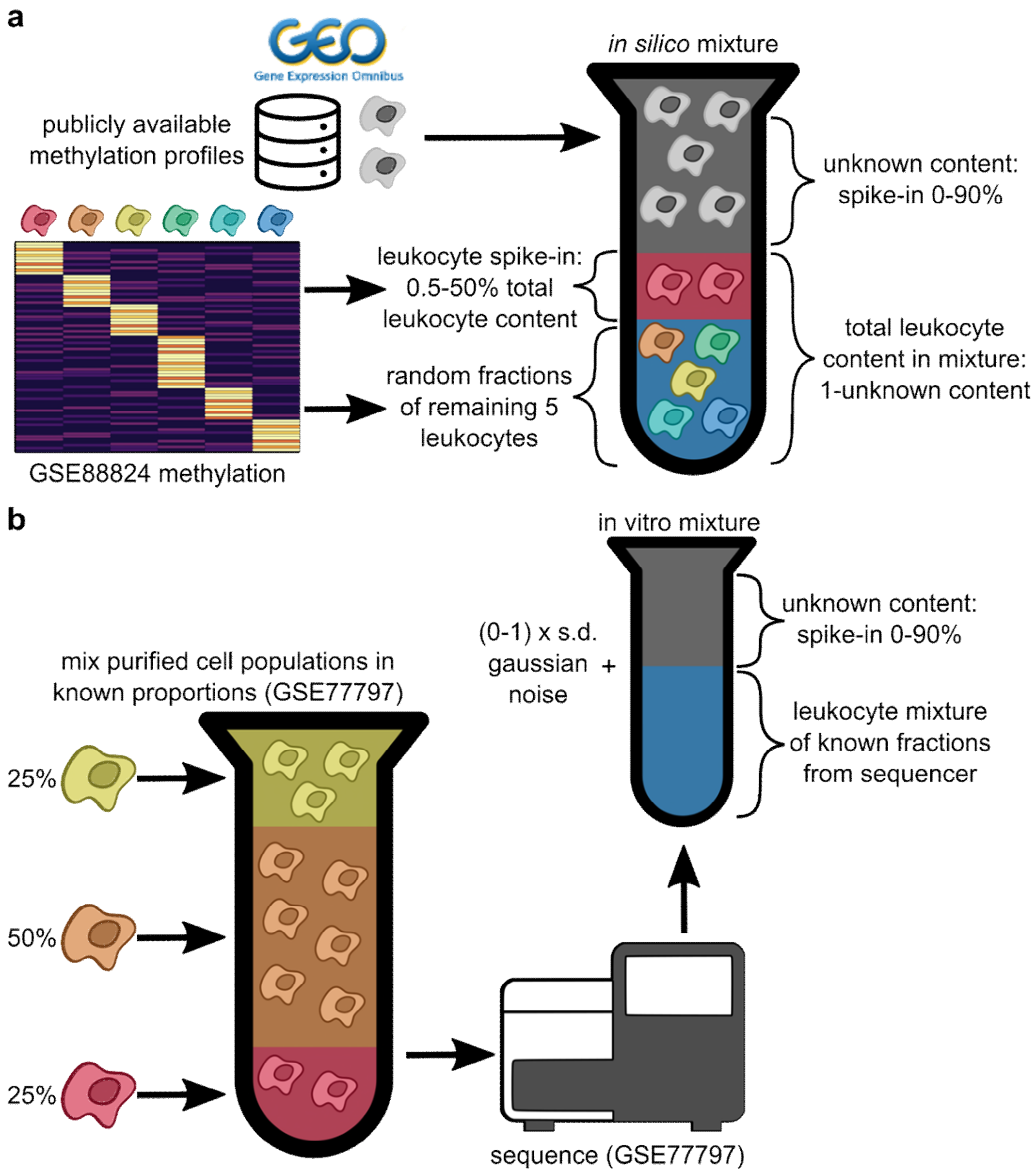


**Supplementary Figure 2.** Schematic of *in silico* and *in vitro* mixture construction for method benchmarking. (**a**). *in silico* mixtures were generated by mixing 6 purified immune cell profiles directly from the IDOL signature matrix. For each mixture, the total immune cell content was comprised of one immune cell type at a known fraction (0.5-50% of total immune cell content) and the rest randomly assigned to the 5 other immune cell types. Additionally, unknown content in the form of cancer cell lines which were not present in the signature matrix were spiked in (0-90% of the total mixture content). (**b**). Immune cell mixtures were obtained from GSE77797 where purified immune cell populations were mixed in known proportions and then sequenced. We generated *in vitro* mixtures by adding unknown content in the form of cancer cell lines (0-90% of the total mixture content) and gaussian noise (0-1 x CpG standard deviation) to the sequenced mixtures.

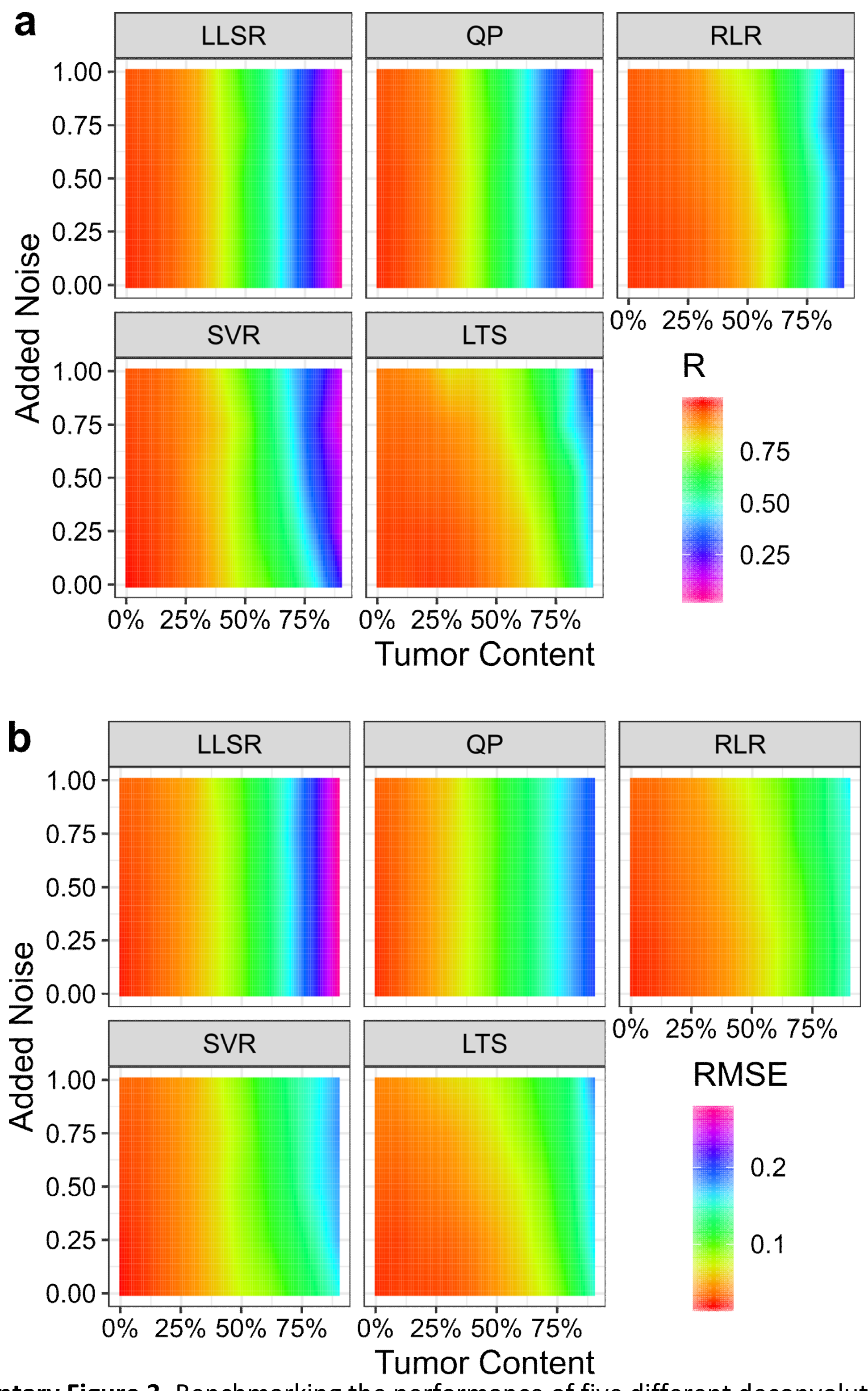


**Supplementary Figure 3.** Benchmarking the performance of five different deconvolution methods using *in vitro* spike-in experiment. The color in the heatmap corresponds to the difference in the Pearson correlation (**a**) or the RMSE (**b**) between the method deconvolution prediction and the ground truth. The y-axis corresponds to the amount of noise that is added, and the x-axis corresponds to the amount of unknown/tumor content in the mixture.

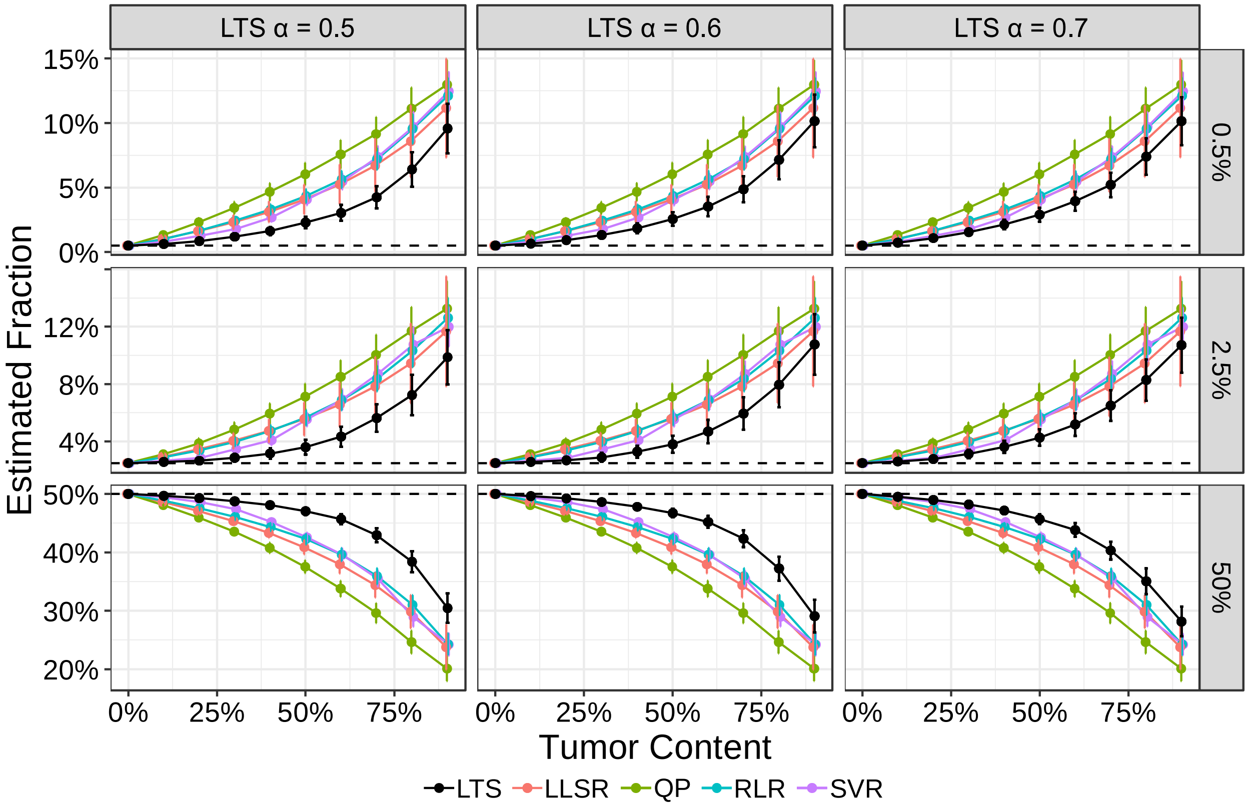


**Supplementary Figure 4.** Benchmarking three different alpha values for use in LTS regression versus four deconvolution methods. Line color corresponds to the deconvolution method, y-axis is the estimated leukocyte fraction, x-axis is the amount of unknown/tumor content in the mixture, columns are the alpha value used for LTS regression, and rows correspond to the known spike-in amount of a particular leukocyte with the dotted line being the ground truth. Error bars are the SEM.

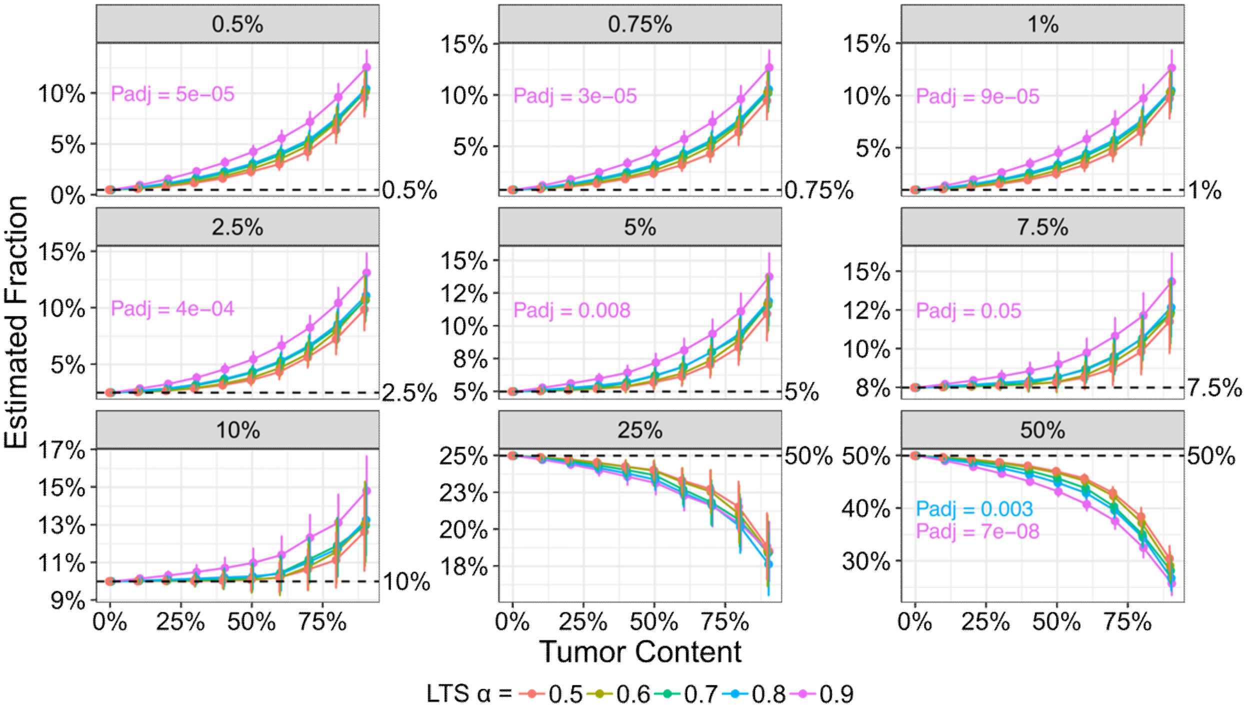


**Supplementary Figure 5.** Benchmarking the choice of alpha parameter in LTS regression. Line color represents the alpha value, y-axis is the estimated leukocyte fraction, x-axis is the amount of unknown/tumor content in the mixture, and each panel corresponds to the known spike-in amount of a particular leukocyte with the dotted line being the ground truth. Statistical significance of the performance differences between all alpha values was determined using post-hoc pairwise comparisons of two-way ANOVA and significant differences between alpha=0.5 and other alpha values were indicated with the color of the text corresponding to the alpha value. Error bars are the SEM.

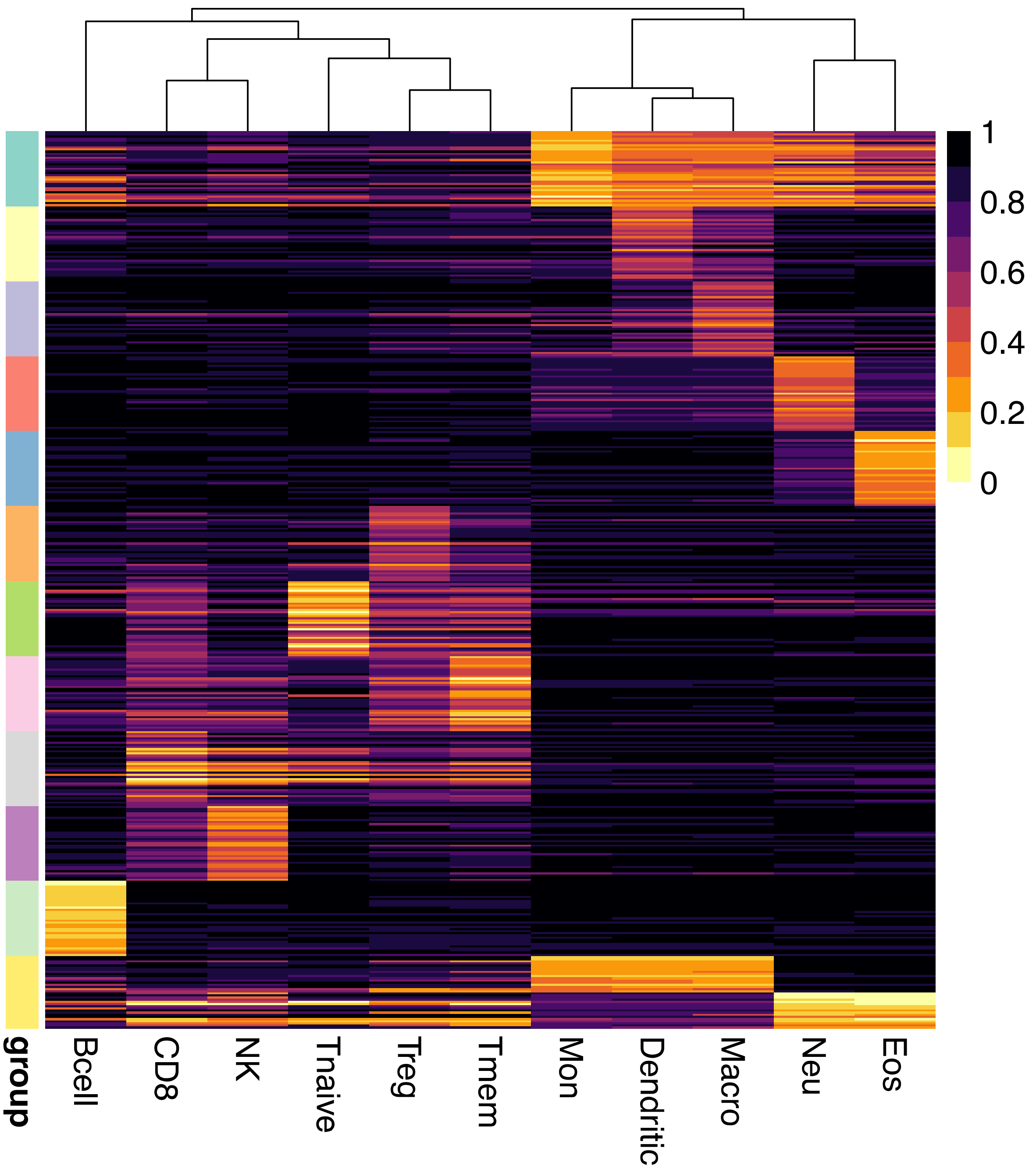


**Supplementary Figure 6.** MethylResolver leukocyte methylation deconvolution reference signature matrix. Color on heatmap corresponds to Beta values, with 35 CpGs per cell type and 34 CpGs to delineate granulocytes from monocytes, dendritic cells, and macrophages. Cell types (columns) are clustered by hierarchical clustering and CpGs in rows are ordered by cell type.

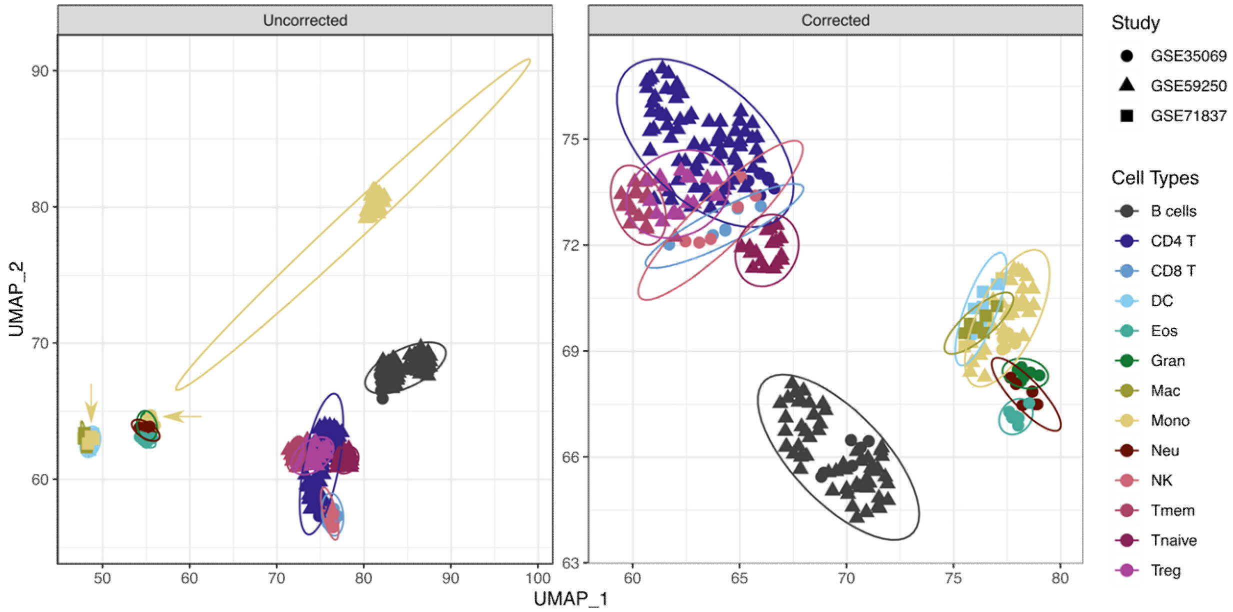


**Supplementary Figure 7.** Batch correction of three purified leukocyte methylation profiling studies which were used to construct the MethylResolver signature matrix. Shape of points corresponds to the study origin and color of points corresponds to cell types. Prior to batch correction, samples were clustering by study, rather than cell type. After batch correction, samples correctly cluster by cell type. Arrows indicate monocytes which cluster separately by batch.

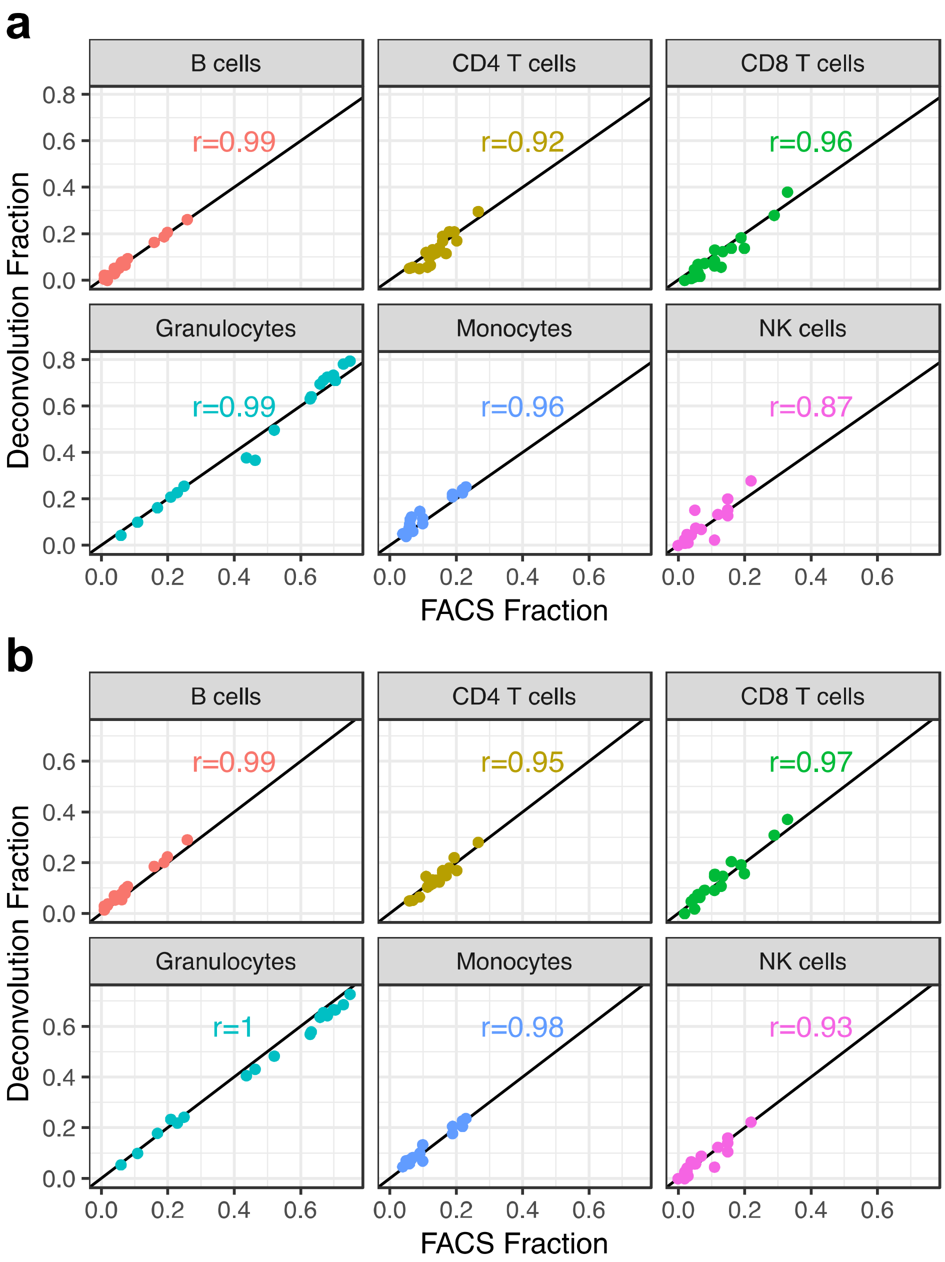


**Supplementary Figure 8.** MethylResolver predicted relative leukocyte fractions (y-axis) using (**a**) our MethylResolver signature matrix and (**b**) the IDOL signature matrix of 12 samples from reconstructed mixtures of purified human leukocytes and 6 samples from adult human whole blood samples with corresponding FACS fractions (x-axis). Each panel gives one of the 6 different cell types in the mixtures or which was quantified in the FACS analysis of whole blood. The Pearson correlation is indicated.

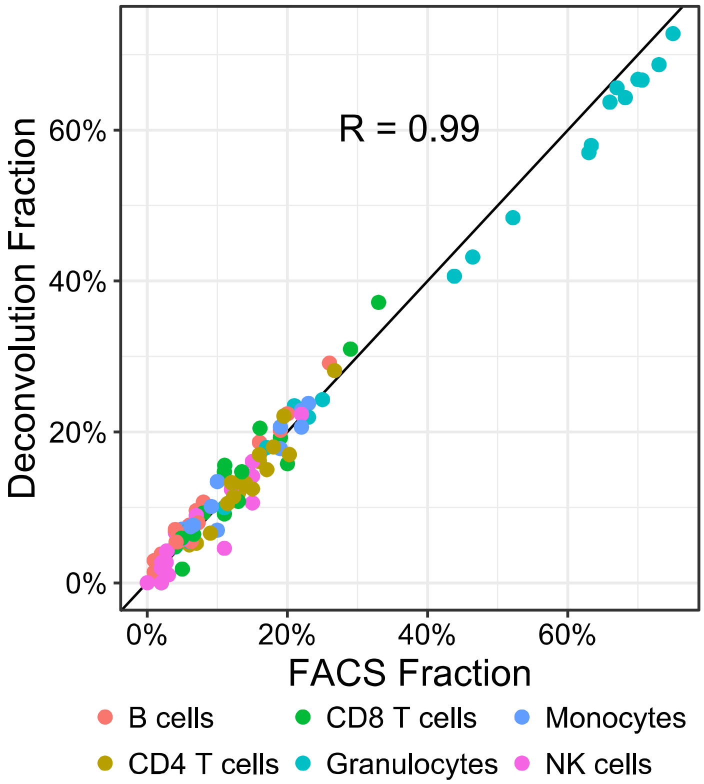


**Supplementary Figure 9.** Performance of IDOL signature matrix on deconvolution of known mixtures and whole blood. Using the IDOL signature matrix, MethylResolver predicted relative leukocyte subset fractions (y-axis) of 12 samples from reconstructed mixtures of purified human leukocytes and 6 samples from adult human whole blood with corresponding FACS fractions (x-axis). Cell type is denoted by the color of each point and Pearson Correlation is indicated.

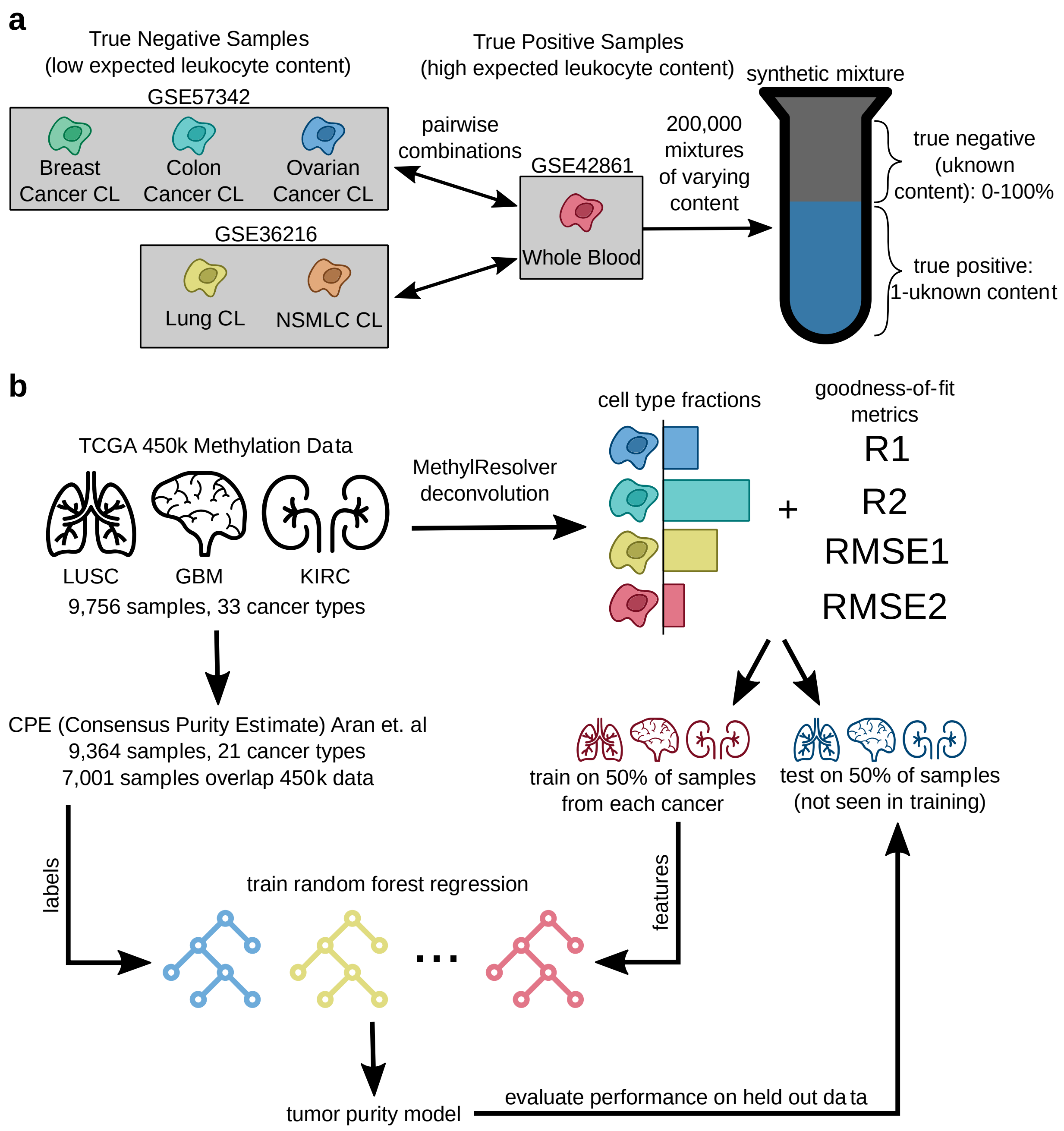


**Supplementary Figure 10.** Schematic of significant deconvolution benchmark construction and tumor purity prediction model. (**a**). To evaluate significant deconvolutions, a benchmark dataset of 200,000 mixtures of varying fractions of low expected leukocyte content samples and high expected leukocyte content samples was generated. (**b**). 7,001 samples with both TCGA 450k methylation data and CPE tumor purity estimates available were deconvolved using MethylResolver. For each cancer type, half of the samples were used to train a random forest regression to predict the CPE tumor purity using the cell type fractions and goodness-of-fit metrics from MethylResolver as features. The resulting model was evaluated on the held-out data which was not seen in the training of the model.

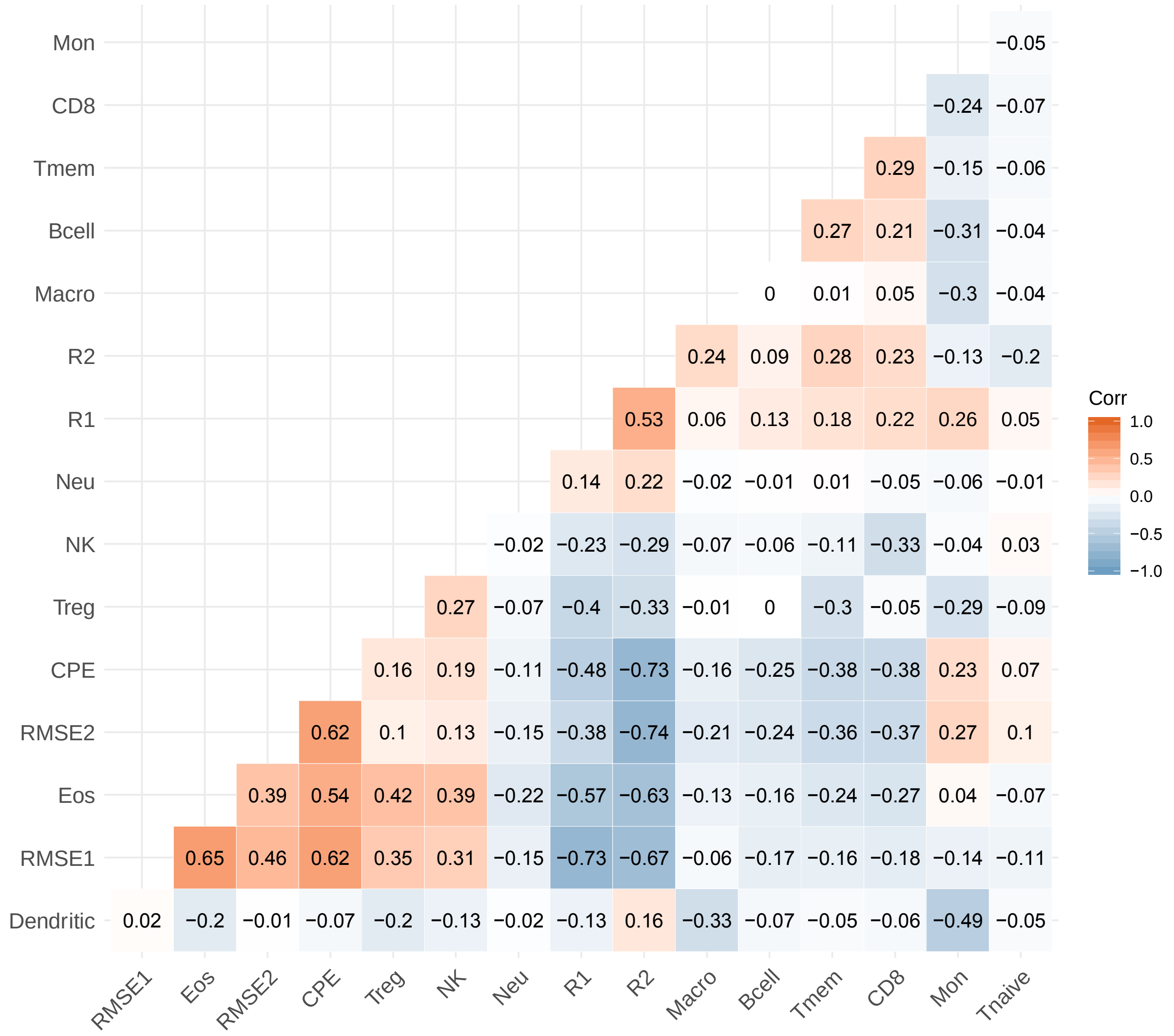


**Supplementary Figure 11**. Pearson correlation of all features in RF regression model to predict tumor purity with CPE tumor purity across 7,001 TCGA cancer samples with available CPE tumor purity estimates.

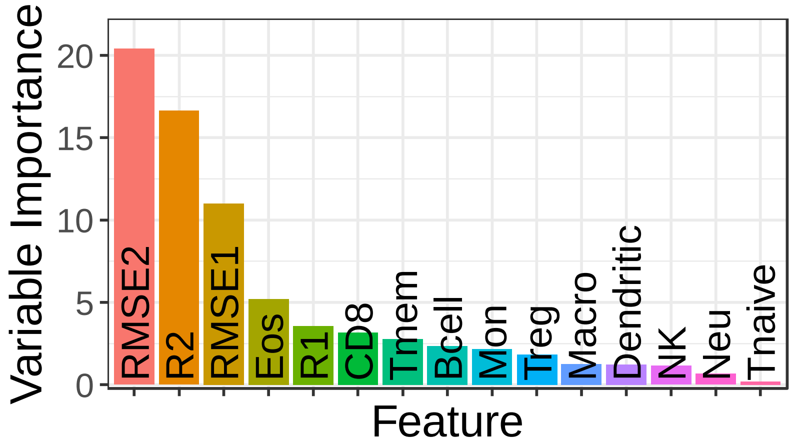


**Supplementary Figure 12**. Variable importance of the features included in the RF regression model to predict tumor purity based on CPE tumor purity values of TCGA cancer samples.

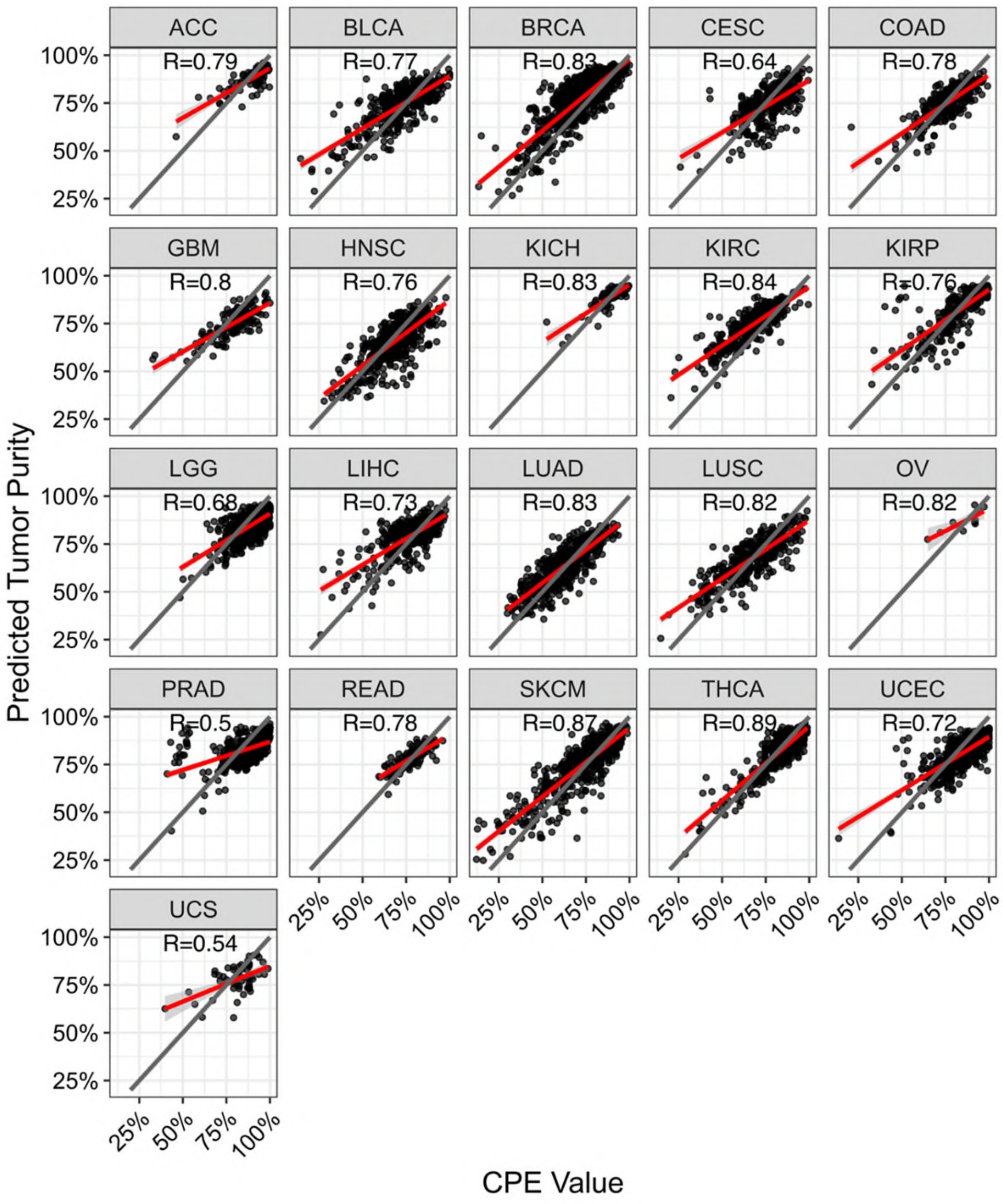


**Supplementary Figure 13**. Performance of MethylResolver tumor purity estimation in held out cancer types. Correlation between MethylResolver predicted tumor purity from our RF regression model (y-axis) and the ground truth CPE tumor purity value (x-axis) for 21 different cancer types from TCGA (panels) with the Pearson correlation indicated. The grey line is y=x and the red line is a linear regression of the data points. Here, the model was trained on 20 of the 21 cancer types and tumor purity was predicted on the held out cancer type for each of the 21 cancer types to obtain an estimate of tumor purity prediction performance on a cancer type which was not seen in training.

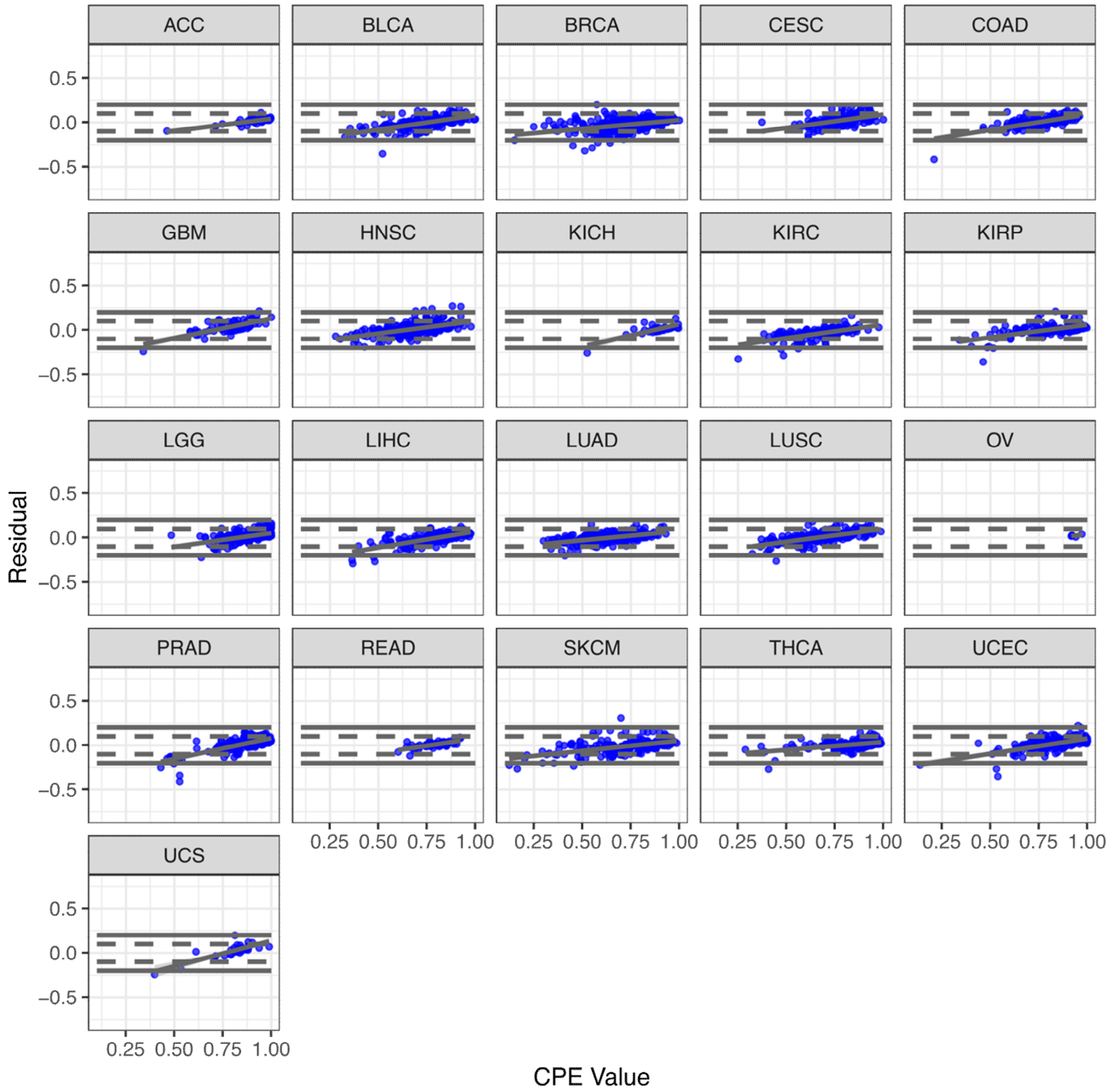


**Supplementary Figure 14**. Residual value (CPE tumor purity estimate – RF regression model tumor purity estimate) of predicted tumor purity for cancer samples from TCGA across 21 different cancer types. These cancer samples used for prediction here were not seen in the training of the RF regression model and all HNSC cancer samples were held out from training of the model. The dashed horizontal lines are +/- 0.1 and the solid horizontal lines +/- 0.2. Each panel contains samples from a different cancer type.

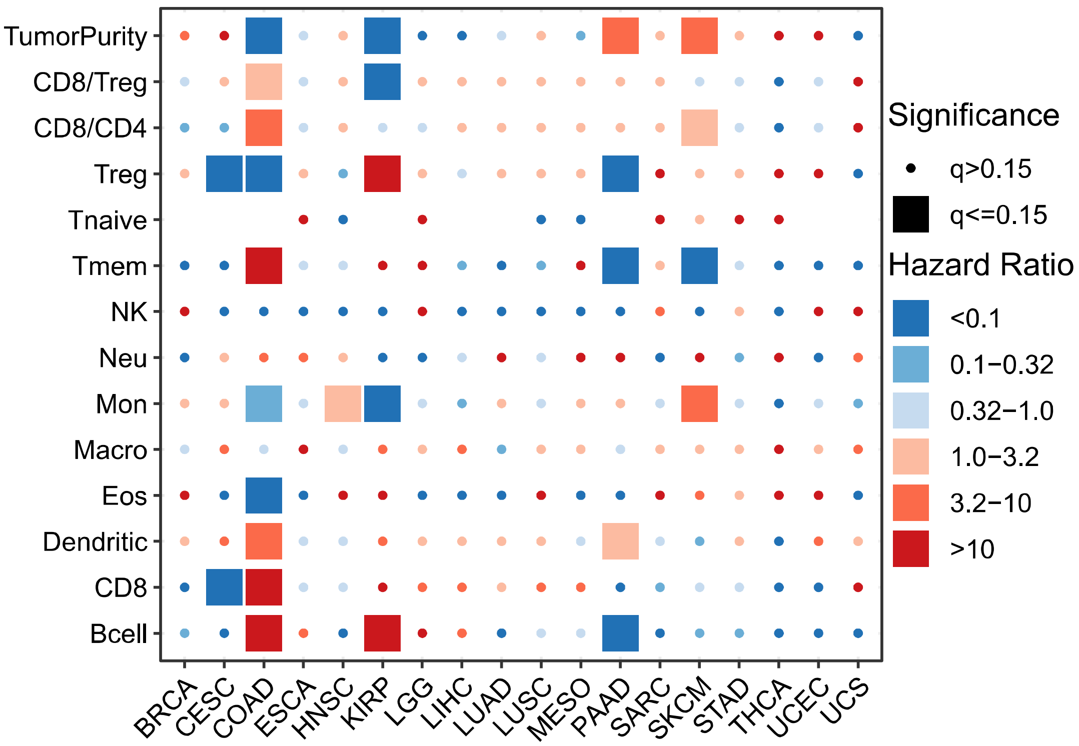


**Supplementary Figure 15.** Prognostic associations of relative leukocyte fractions from pan-cancer deconvolution. Multivariate Cox regression was applied to the MethylResolver pan-cancer deconvolution of TCGA to infer prognostic leukocytes using relative fractions from significant deconvolutions. Heatmap colors correspond to the hazard ratio values and shapes correspond to the significance, rows correspond to cancer type and columns correspond to cell type, tumor purity, or neutrophil-to-lymphocyte ratio (NLR).
